# Working Adults' Intentions to Participate in Microlearning: Assessing for Measurement Invariance and Structural Invariance

**DOI:** 10.3389/fpsyg.2021.759181

**Published:** 2021-11-29

**Authors:** Shermain Puah, Muhammad Iskandar Shah Bin Mohmad Khalid, Chee Kit Looi, Ean Teng Khor

**Affiliations:** ^1^Centre for Research and Development in Learning, Nanyang Technological University, Singapore, Singapore; ^2^National Institute of Education, Nanyang Technological University, Singapore, Singapore

**Keywords:** decomposed theory of planned behaviour, technology acceptance, adult learning, measurement invariance, microlearning, structural invariance

## Abstract

The current study set out to understand the factors that explain working adults' microlearning usage intentions using the Decomposed Theory of Planned Behaviour (DTPB). Specifically, the authors were interested in differences, if any, in the factors that explained microlearning acceptance across gender, age and proficiency in technology. 628 working adults gave their responses to a 46-item, self-rated, 5-point Likert scale developed to measure 12 constructs of the DTPB model. Results of this study revealed that a 12-factor model was valid in explaining microlearning usage intentions of all working adults, regardless of demographic differences. Tests for measurement invariance showed support for invariance in model structure (configural invariance), factor loadings (metric invariance), item intercepts (scalar invariance), and item residuals (strict invariance) between males and females, between working adults below 40 years and above 40 years, and between working adults with lower technology proficiency and higher technology proficiency levels. While measurement invariance existed in the data, structural invariance was only found across gender, not age and technology proficiency. We then assessed latent mean differences and structural path differences across groups. Our findings suggest that a tailored approach to encourage the use of microlearning is needed to suit different demographics of working adults. The current study discusses the implications of the findings on the use and adoption of microlearning and proposes future research possibilities.

## Introduction

In today's fast-paced and technology-driven working environment, expectations of lifelong learning for many working professionals are shifting toward just-in-time instruction and on-need training (Brandenburg and Ellinger, 2003). *Microlearning* fulfils these expectations as it emphasises delivering self-paced bite-size content and just-in-time training (Hug and Friesen, 2007). As learners are also opting to exercise greater control over their intentions and actual usage of technology for professional development, the microlearning design helps cultivate a lifelong learning culture where people effectively manage their own learning independently in various contexts (Sharples, 2000). With greater volition over technology use, it is important to address the factors driving greater intentions to use microlearning, and understand if these factors differ across working adults of different demographics. The current study investigates differences in working adults' intentions to participate in microlearning using the decomposed theory of planned behaviour (DTPB) across gender, age, and proficiency in technology.

### Microlearning

Hug (2015) defines microlearning as “an abbreviated manner of expression for all sorts of short-time learning activities with microcontent” (p. 492). Similarly, Shail (2019) defines it as “relatively small, focused learning units consisting of condensed learning activities (usually one to 10 min), available on multiple devices” (p. 2). For busy working professionals, traditional e-Learning are no longer attractive or effective. Instead, microlearning formats, where training content delivered in bite-size chunks that may be accessed from anywhere and at any time, are increasingly being considered for use in professional development.

### Decomposed Theory of Planned Behaviour (DTPB)

Taylor and Todd (1995) introduced the DTPB, which includes not only the core constructs of the theory of planned behaviour (TPB; Attitude, Subjective Norms, Perceived Behavioural Control), but an exhaustive set of antecedents as decomposed constructs, to comprehensively capture intentions toward technology adoption. Decomposing the belief structure of the TPB was thought to increase explanatory power of the model and may help in greater understanding of the determinants of behavioural intentions. The authors compared several competing technology adoption models for predicting behavioural intentions to use information technology, and reported the DTPB as being superior in terms of overall model fit, explanatory power, and insights drawn about behavioural intentions.

**Figure 1:**
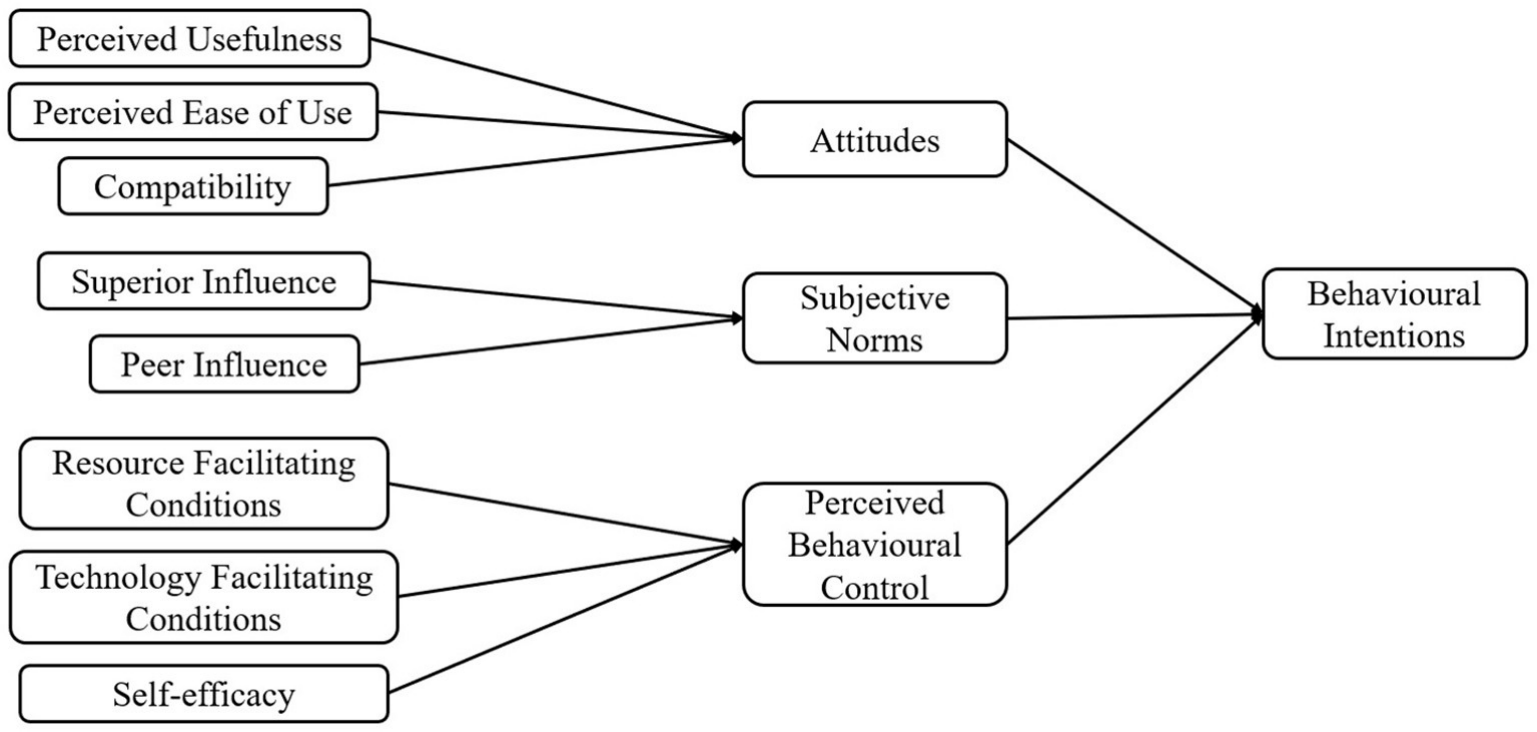
DTPB model used to study working adults' microlearning usage intentions (Taylor and Todd, 1995).

Therefore, using the DTPB, the current study investigates the factors influencing microlearning usage intentions in working adults, and more importantly, explores if the strength of the relationships between the factors vary according to age, gender, and technology proficiency. The better explanatory power of the DTPB may afford a more detailed and satisfactory account of behavioural intentions for a fairly new learning technology like microlearning, and thus facilitate and support its adoption in our workforce. Figure 1 presents the adapted DTPB model explored in this study.

### Attitude

Attitude refers to one's positive or negative feelings toward a behaviour; more positive Attitude usually results in stronger behavioural intentions. Attitude can be decomposed into Perceived Usefulness (PU), Perceived Ease of Use (PEU), and Compatibility. PU refers to “the degree to which a person believes that a particular technology would enhance his or her job performance” (Davis, 1989, p. 320), PEU refers to “the degree to which a person believes that the utilisation of a particular technology would be free of effort” (Davis, 1989, p. 320), while Compatibility refers to the fit of one's existing needs, goals, and experiences with a new technology (Rogers, 1983). In general, greater PU, PEU, and Compatibility leads to more positive attitudes toward technology adoption.

### Subjective Norms

Subjective Norms (SN) refer to perceived expectations from important others' that influence one's intentions to perform a specific behaviour (Fishbein and Ajzen, 1975). Decomposing SN forms two referent groups that are said to heavily influence one's normative beliefs; they are Peer Influence (PI) and Superior Influence (SI). “Peers” would refer to those in the same social circle as oneself, while “superiors” are those in positions of authority at one's place of work. Both groups may have opposing opinions regarding technology adoption (i.e., supportive vs. non-supportive), and thus have to be considered separately (Taylor and Todd, 1995).

### Perceived Behavioural Control

Perceived Behavioural Control (PBC), which accounts for situations where one does not have control over their behaviour, is characterised by perceptions of internal and external constraints on one's behaviour (Taylor and Todd, 1995). PBC can be decomposed into Self-efficacy (SE), Resource Facilitating Conditions (RFC), and Technology Facilitating Conditions (TFC). SE reflect one's judgment of their own ability to perform specific behaviours, while RFC and TFC reflect the availability of resources required to perform the behaviour (e.g. time, money, technological equipment). Greater SE results in greater intentions to perform a behaviour, whereas the lack of facilitating conditions can decrease intentions to adopt a new technology.

### Behavioural Intentions

Posited as the main contributing factor of actual behaviour, behavioural intention has been found notably useful for predicting different behaviours (Sheppard et al., 1988). However, a long-standing debate exists with regards to the actual strength of the relationship between intentions and behaviour. Previous meta-analyses indicate that intentions do largely translate into actual behaviour (e.g., Webb et al., 2006; Sheeran and Webb, 2016). For example, Sheeran (2002) meta-analysis of previous meta-analyses (involving 422 studies in total) found a large correlation (*r* = 0.53) between intentions at one time-point and actual behaviour measured at a later time-point. More recently, Wood et al. (2016) meta-analysed 116 studies and reported a significant, albeit small, positive effect (*d* = 0.24) of asking intentions and self-prediction questions on one's subsequent behaviours.

### Moderating Role of Individual Differences

As opposed to the use of microlearning with homogeneous populations, such as undergraduates who typically present similar demographics, studying the use of microlearning with the broader workforce requires a more cautious approach as individual differences may have profound influences on one's beliefs and intentions. Although studies have found that Attitude, SN, and PBC directly and positively affect intentions about using e-Learning (e.g., Hung et al., 2016), the extent to which the relationships between various constructs, and the influence of these constructs on one's intentions to participate in microlearning varies, due to age, gender, and proficiency in technology, remains unclear.

Differences amongst adults may never cease to exist in our working society, making it imperative to understand what drives differences in working adults' intentions to use microlearning, such that measures can be taken to address different responses to adopting a new technology in various groups of people. This ensures that the implementation of microlearning at various organisations or adult learning institutions will not be underused by certain populations or not used at all. To the best of our knowledge, no study has explored the moderating effects of demographic differences in microlearning adoption with the DTPB.

Drawing from past studies (e.g., Morris and Venkatesh, 2000; Venkatesh et al., 2000; Morris et al., 2005) that have investigated the influence of user differences using similar intentions-based models, such as the TPB, we may be able to make inferences about the way these individual differences will manifest in influencing the relationships between the decomposed TPB constructs and intentions. For instance, Venkatesh et al. (2000) applied the TPB model to examine gender as a moderator of the determinants of workers' intentions and use of a new software application over a 5-month period. The authors found gender differences only at the first month mark, with males more influenced by Attitude toward using the software, and females more heavily influenced by social factors (i.e., SN) and environmental constraints (i.e., PBC). By the second month, these gender differences in determining software use were non-significant.

In terms of the moderating effects of age, Morris and Venkatesh (2000) reported that intentions about short-term software use were influenced by Attitude, SN, and PBC in older working adults, while younger working adults were solely influenced by Attitude toward using the software. The influence of SN on older working adults' intentions to use technology became non-significant when making long-term decisions; over time, decisions made regarding technology usage shifted from compliance to others' opinion to independent assessments for whether they should use that technology. PBC remains an important factor influencing software use intentions in older workers as high importance is placed on receiving support and assistance as they continue to use the technology. Therefore, with a highly diverse group of working adults, demographic characteristics should be studied alongside determinants of behavioural intentions to provide a more comprehensive view of whether one decides to use microlearning or not.

### Testing for Measurement Invariance

However, research comparing across groups of adults differing in various demographics, typically assume the equivalence of responses and rarely verify if that assumption is accurate (Teo, 2015, 2019). If a measure is not sufficiently invariant across groups, it increases risk for making spurious conclusions of between-group differences based on biassed estimates that have considerable error. Testing for measurement and structural invariance prior to making comparisons across groups prevents such situations from occurring (Byrne et al., 1989; Vandenberg and Lance, 2000; Byrne and Stewart, 2006). On the other hand, if the assumption of measurement invariance is supported, any subsequent interpretations of between-group differences can be definitively attributed to true differences in path coefficients, not to measurement-related differences.

To our knowledge, this has rarely been done in technology adoption studies. One example includes the study by Teo (2019) which used several constructs from intentions-based models to assess measurement and structural invariance of the model for explaining students' and teachers' intention to use computers and technology. Full configural and metric invariance was met, and partial scalar invariance was found in their data. Structural factor invariance was supported by only two out of nine regression paths; a positive influence of PU on Attitude and a negative influence of computer SE on Attitude were found to be equally applicable to students and teachers. This implies that many of the relationships between factors have different impacts depending on whether one is a student or teacher.

Previously, Teo (2015) assessed for measurement invariance between pre-service and in-service teachers' intentions to adopt technology; these findings then allowed them to analyse latent mean differences between these two groups. Tests of measurement invariance showed support for scalar invariance (i.e., most restrictive model) for five variables (PU, PEU, Attitude, SN, and SE), and latent mean comparisons thereafter revealed that pre-service teachers endorsed these variables higher than in-service teachers though these differences were not found to be significant.

### Present Study

In the present study, we use the DTPB as a framework to study and identify the factors that influence working professionals' intentions to participate in microlearning. Specifically, we examine if the DTPB constructs themselves and the relationships between constructs differ between males and females, between working adults from different age groups (i.e., working adults below 40 years vs. above 40 years^1^), and between working adults with different technology proficiency levels (i.e., moderately proficient and below vs. highly proficient and above). This would ascertain if any observed differences were due to actual gender, age, and technology proficiency differences, or if the differences are a result of non-invariant measurement structures. Findings will contribute to greater understanding of the feasibility of applying DTPB to study microlearning adoption and will reflect the importance of considering learners' intentions to participate in microlearning. Findings from this study has practical implications for how to promote the use and adoption of microlearning in the workplace based on one's gender, age, working experience, and proficiency in technology. Thus, we ask the following exploratory research questions:

1. Are the constructs of the DTPB model *measurement invariant* across gender, age, and levels of technology proficiency?
2. Is the DTPB model *structure invariant* across gender, age, and levels of technology proficiency?
3. Are there significant latent mean differences between groups of adults of differing gender, age, and technology proficiency for each factor in the DTPB model?
4. Are there significant structural path (i.e., regression coefficient) differences between groups of adults of differing gender, age, and technology proficiency for each factor in the DTPB model?

## Methods

### Sample

A total of 628 working adults from Singapore participated in our online study (306 females, 322 males; mean age group^2^ females = 1.65, *SD* = 0.94, mean age group males = 1.83, *SD* = 0.99). Participants had to be at least 17-years-old, and have prior working experience and e-Learning experience. They were either recruited from adult learning institutes or got to know of the study through word-of-mouth, and took part on a voluntary basis. As a result of the COVID-19 restrictions, the authors were unable to proceed with physical data collection; this might have biassed the participant sample toward working adults who are better versed with digital technology and thus is not an accurate representation of the working population of Singapore.

Our sample size was determined using a general rule of thumb which found a sample size of 200 or more adequate for a SEM analysis (Kline, 2015). We aimed to collect at least 500 responses between the period of October 2020 to December 2020, and eventually collected more data than expected. Since previous research studying behavioural intentions of e-Learning usage using the DTPB model reported sample sizes ranging between 200 and 500 (Ahmed and Ward, 2016; Renda dos Santos and Okazaki, 2016), our sample size of 628 is sufficiently larger than previous samples.

Table 1 includes demographic data of the participants, and the comparison groups they were grouped into for the measurement invariance analyses. These data-informed splits were made to maintain high robustness in our results; Meade (2005) ran a simulation study and found that a sample size of 100 per group was insufficient, and produced less robust results than sample sizes of 200 and 400. As such, we made a logical split such that each comparison group had at least 200 data points. To this end, working adults aged between 17 to 39 were grouped together (*n* = 395) and those 40 and above grouped into the second group (*n* = 233); also, working adults who rated themselves as “Not proficient at all,” “Slightly proficient,” and “Moderately Proficient” were grouped together (*n* = 204), while those who rated themselves as “Highly Proficient” and “Extremely Proficient” were grouped together (*n* = 424). We acknowledge that in recategorising age and technology proficiency groups into wider groups, due to the lack of sufficient data points to categorise participants into more distinct groups, we lost some valuable information.

**Table 1:** Demographics of participants.

**Variable**		***N***	**Comparison Group**
Gender			
	Males	322	0
	Females	306	1
Age group			
	17–29 years	226	0
	30–39 years	169	
	40–49 years	104	1
	50–59 years	94	
	60–69 years	34	
	70 years and above	1	
Technology proficiency			
	Not proficient at all	3	0
	Slightly proficient	29	
	Moderately proficient	172	
	Highly proficient	285	1
	Extremely proficient	139	

### Measures

An author-developed instrument consisting of multiple 5-point Likert scale items measuring the 12 constructs of the DTPB model in Figure 1 was created by adapting from previous published sources (e.g., Davis, 1989; Taylor and Todd, 1995). In addition to demographic questions and microlearning knowledge questions, 46 items were used as a measure of participants' responses to 12 constructs: Attitude (3 items); PU (4 items); PEU (4 items); Compatibility (4 items); SN (4 items); PI (4 items); SI (4 items); PBC (4 items); SE (4 items); RFC (4 items); TFC (4 items); and behavioural intentions (3 items). Appendix A details the full survey instrument used.

Prior to data collection, we pre-tested our initial survey instrument with a small group of participants (*N* = 31). We assessed the internal reliability of our measure using Cronbach's α (Watkins, 2018), and found that coefficient α for all constructs were above 0.70, except for the construct “peer influence” (α = 0.55). To improve internal consistency of the “peer influence” construct, we decided to replace the item having the lowest correlation with all other items (i.e., “I will participate in microlearning if my peers have done/are doing so too.” was replaced by “The opinions of my peers, for whether I participate in microlearning, is important to me.”). Our final scale showed satisfactory α coefficients ranging from 0.78 to 0.92, implying adequate internal consistency amongst items (Watkins, 2018). Table 2 shows Cronbach's α estimates for the pretested scale and final scale used.

**Table 2:** Pretest and final study scale reliability using Cronbach's α.

**Construct**	**No. of items**	**α Coefficient (Pretest)**	**α Coefficient (Final)**
Attitude	3	0.90	0.87
PU	4	0.94	0.87
PEU	4	0.74	0.78
Compatibility	4	0.93	0.91
SN	4	0.88	0.83
PI	4	0.55	0.78
SI	4	0.89	0.92
PBC	4	0.91	0.84
SE	4	0.90	0.86
RFC	4	0.81	0.82
TFC	4	0.83	0.79
Intentions	3	0.90	0.90

### Data Analyses

Statistical analyses were conducted with the R 4.0.3 software (R Core Team., 2020). SEM was performed using the *lavaan* (Rosseel et al., 2020) package, while measurement invariance analysis was conducted with *purrr* (Henry et al., 2020), and *semTools* (Jorgensen et al., 2021) packages. Reproducible R-scripts and data files for our analyses can be found here: https://doi.org/10.21979/N9/NZ8N7M.

### Measurement Invariance

Multigroup invariance analyses were conducted to test for measurement (configural, metric, scalar, and strict) and structural (factor variances and covariances) invariance between males and females, between working adults from different age groups (i.e., working adults below 40 years vs. above 40 years), and between working adults with different technology proficiency levels (i.e., moderately proficient and below vs. highly proficient and above). Testing for multigroup invariance of structural equation modelling (SEM) models allows one to explicitly test the equivalence of the model structure or individual coefficients across multiple groups by addressing the measurement invariance (configural, metric, scalar, and strict invariance) and structural invariance (factor variances and covariances invariance) of the model (Byrne et al., 1989; Vandenberg and Lance, 2000; Byrne and Stewart, 2006). By establishing measurement invariance, we ensured that the data is adequately invariant to allow for any between-group comparisons to be made.

### Order of Invariance Analysis

Assessing multigroup invariance involves a sequential ordering of nested models with the less constrained model being used as a criterion for evaluation of the more constrained model. The difference between the less constrained model nested within the more constrained model can be assessed with a chi-square test of difference (Δχ^2^). However, researchers (Cheung and Rensvold, 2002; Chen, 2007) highlighted that Δχ^2^ is still very much affected by large sample sizes and deviations from normality, leading to unrealistic and improbable standards for showing evidence of invariance.

Therefore, alternative criteria should be used to assess for evidence of measurement invariance, namely (a) adequate overall model fit of the multigroup model based on cut-off recommendations of fit indexes (Byrne and Stewart, 2006); and (b) a non-significant change in goodness-of-fit indexes between nested models as an indication that observed differences may be explained by chance [i.e., ΔCFI ≤ 0.01 (Cheung and Rensvold, 2002), ΔRMSEA ≤ 0.015, ΔSRMR ≤ 0.01 (Chen, 2007)]. As recommended by Hu and Bentler (1999), a model shows acceptable fit when it fulfils the criteria of having a comparative fit index (CFI) of above 0.90 (while CFI of 0.95 and above signifies good model-data fit), root mean square error of approximation (RMSEA) of 0.06 or lower, and standardised root mean residual (SRMR) of 0.08 or lower.

#### Configural Invariance

The baseline model, to which subsequent more restrictive models are compared against, is one that supports configural invariance. Specifically, the model that produces the best fit to the data for each group forms the baseline model, and configural invariance is established if the number of factors and factor loading patterns are invariant across groups.

#### Measurement Invariance

*Metric invariance* (or weak measurement invariance) is assessed by constraining factor loadings of items to be invariant across groups in the SEM model. A non-significant change in goodness-of-fit between this model and the baseline model indicates that the relationships between latent variables and its respective observed items are equivalent across all groups, hence supporting metric invariance.

Next, *scalar invariance* (or strong measurement invariance) is tested by constraining both factor loadings and intercepts of items to be invariant across groups. Establishing scalar invariance implies that the starting point of the scale is equivalent for all groups, hence allowing a fair comparison of factor means across groups. In contrast, failing to satisfy scalar invariance points to the existence of measurement bias (or differential item functioning), where a group-specific norm causes individuals from that group to systematically give higher or lower ratings on item(s) of a construct, in turn resulting in latent mean differences between groups.

Finally, *strict invariance* is tested by constraining residuals of observed items, that may be attributed to both random measurement error and error due to specific measurement properties, to be invariant across groups (Vandenberg and Lance, 2000). As a result, differences between groups, if any, would solely be due to true group differences on the latent constructs. However, strict invariance has been said to be very difficult to obtain and is also not required for making meaningful latent mean comparisons.

#### Structural Invariance

Tests of factor variance and covariance invariance are treated as omnibus tests of whether specified causal relationships among latent constructs fit equally well across groups (Vandenberg and Lance, 2000). The invariance properties of these structural parameters are only tested when the invariance of the parameters of the measurement model was supported. Thereafter, the model with factor covariances and variances constrained to be invariant across groups was compared against the baseline model with configural invariance held. Support for the invariance of factor variances and covariances would imply that the structural model imposed on the latent constructs are equivalent across groups (i.e., a lack of group difference in structural relationships). In contrast, a significant change in goodness-of-fit between these models would then indicate that at least some differences in structural path coefficients between groups likely exist. In that case, the invariance of each structural parameter (or regression coefficient) in the factor variance and covariance matrix is tested.

## Results

Prior to conducting multigroup invariance analysis, we assessed the model-data fit and factor loadings for the full sample (*N* = 628). Then, to make comparisons between groups of participants of differing gender, age groups, and technology proficiency, we first tested for measurement invariance (configural, metric, and scalar) between groups and thereafter, tested the structural invariance of our research model.

### Model-Data Fit

As results from the CFA revealed a poor model-data fit for the full sample (CFI = 0.87; RMSEA = 0.07; SRMR = 0.07). Improvements were made by removing items contributing to poorer measurement quality (i.e., 6 items that had weak factor loadings of less than 0.7, and 4 items that were more strongly correlated with items measuring another factor than its intended factor). The resulting final measurement model with 36 items loaded onto 12 factors produced a satisfactory model-data fit (CFI = 0.95; RMSEA = 0.05; SRMR = 0.04). For more information, supplementary materials include a detailed breakdown of the various analyses carried out at the measurement level to ensure high measurement quality of the measurement model (see online supplementary file https://doi.org/10.21979/N9/NZ8N7M).

### Measurement Invariance

Table 3 includes the results for multigroup invariance of the DTPB model across gender, age group, and technology proficiency. In general, the tests of measurement invariance showed support for invariance in model structure, factor loadings, item intercepts, and item residuals between males and females, between working adults below 40 years and above 40 years, and between working adults with lower technology proficiency and higher technology proficiency levels. According to various fit indexes, the 12-factor solution for each group across gender, age, and technology proficiency showed an acceptable model fit (0.92 ≤ CFIs ≤ 0.93, RMSEAs = 0.06, SRMR = 0.08). Although both RMSEA and SRMR fell exactly on the bounds of cut-off recommendations, and CFI fell close to the cut-off bounds, the existence of a *reliability paradox*, which illustrates how models are punished for having better measurement quality (i.e., large sample size, standardised factor loadings > 0.7) while models with poorer measurement quality achieve good model-data fit more easily, makes an acceptable model-data fit more difficult to obtain in such cases where there is better measurement quality (Heene et al., 2011; McNeish et al., 2017). Therefore, since ensuring a high measurement quality in our measurement model, a slight difference in values of fit indexes from the cut-off recommendations is not necessarily an indication of poor fit.

**Table 3:** Results of measurement invariance analysis.

**Model**	**CFI**	**RMSEA (90% CI)**	**SRMR**	**Model comp**	***df* (Δ*df*)**	**ΔCFI**	**ΔRMSEA**	**ΔSRMR**	**Decision**
***Gender—females (n** **=** **306) and males (n** **=** **322)***									
M1A	0.927	0.059(0.056–0.063)	0.075	–	1,110 (–)	–	–	–	–
M2A	0.927	0.059(0.055–0.062)	0.075	M2A vs. M1A	1,134 (34)	–	–	–	Accept
M3A	0.927	0.058(0.055–0.061)	0.075	M3A vs. M2A	1,158 (24)	–	0.001	–	Accept
M4A	0.917	0.061(0.058–0.064)	0.077	M4A vs. M3A	1,194 (36)	0.010	0.003	0.002	Accept
M5A	0.916	0.060(0.057–0.063)	0.084	M5A vs. M1A	1,234 (124)	0.011	0.001	0.009	Accept
***Age group−40 years and below (n** **=** **395) and above 40 years (n** **=** **233)***									
M1B	0.919	0.063(0.059–0.066)	0.076	–	1,110 (–)	–	–	–	–
M2B	0.919	0.062(0.059–0.065)	0.077	M2B vs. M1B	1,134 (34)	–	0.001	0.001	Accept
M3B	0.916	0.062(0.059–0.066)	0.078	M3B vs. M2B	1,158 (24)	0.003	–	0.001	Accept
M4B	0.908	0.064(0.061–0.068)	0.079	M4B vs. M3B	1,194 (36)	0.008	0.002	0.001	Accept
M5B	0.907	0.064(0.061–0.067)	0.085	M5B vs. M1B	1,234 (124)	0.012	0.001	0.011	**Reject**
***Technology proficiency—Moderately proficient and below (n** **=** **204) and highly proficient and above (n** **=** **424)***									
M1C	0.926	0.059(0.055–0.062)	0.075	–	1,110 (–)	–	–	–	–
M2C	0.926	0.058(0.055–0.061)	0.075	M2C vs. M1C	1,134 (34)	–	0.001	–	Accept
M3C	0.923	0.059(0.055–0.062)	0.076	M3C vs. M2C	1,158 (24)	0.001	0.001	0.001	Accept
M4C	0.913	0.061(0.058–0.065)	0.076	M4C vs. M3C	1,194 (36)	0.010	0.002	–	Accept
M5C	0.910	0.061(0.058–0.065)	0.085	M5C vs. M1C	1,234 (124)	0.016	0.002	0.010	**Reject**

*M1, Configural invariance; M2, Metric invariance; M3, Scalar invariance; M4, Strict invariance; M5, Factor variance and covariance invariance. Only values here are only rounded to 3 d.p to aid with calculating change in fit indexes*.

Next, the test of an invariant pattern of factor loadings was also supported for each group across gender, age, and technology proficiency with no ΔCFI above 0.01, ΔRMSEA above 0.015, or ΔSRMR above 0.01 between any Model 2 and Model 1. Then, with the scalar invariance test constraining item intercepts to be equivalent for each group across gender, age, and technology proficiency being established, with no ΔCFI above 0.01, ΔRMSEA above 0.015, or ΔSRMR above 0.01 between any Model 3 and Model 2, strong measurement invariance was supported. Finally, although not necessary for comparing latent mean differences, an invariant pattern of item residuals was tested by constraining all error variances of observed items to be equivalent for each group across gender, age, and technology proficiency; the test of an invariant pattern of item residuals was tenable (i.e., ΔCFI ≤ 0.01, ΔRMSEA ≤ 0.015, ΔSRMR ≤ 0.01).

### Latent Mean Differences

With the invariance of the factor loadings and intercepts supported (i.e., full scalar invariance), it was then possible to make comparisons of the latent mean differences across groups for gender, age, and technology proficiency (see Table 4). Latent mean comparisons were done by constraining one group's latent means to 0 (i.e., males, working adults below 40 years, less technologically proficient) and allowing the latent means of the other group to be freely estimated. Critical ratio (CR) values, calculated as a ratio of the parameter estimate to its standard error, were then used to assess for the presence of latent mean differences. CR values greater than +/−1.96 were indicative of a statistically significant difference between the reference group and comparison group.

**Table 4:** Latent mean differences.

**Construct**	**Comparison groups**		**CR**	***SE***
Attitude	Males	Females		
	0	1	−0.09	0.03
	40 and below	Above 40		
	0	1	0.22	0.03
	Less technology proficient	More technology proficient		
	0	1	−0.54	0.04
PU	Males	Females		
	0	1	−0.69	0.06
	40 and below	Above 40		
	0	1	1.30	0.06
	Less technology proficient	More technology proficient		
	0	1	**2.22**	0.06
PEU	Males	Females		
	0	1	0.41	0.05
	40 and below	Above 40		
	0	1	−0.20	0.06
	Less technology proficient	More technology proficient		
	0	1	**5.70**	0.06
Compatibility	Males	Females		
	0	1	−0.12	0.06
	40 and below	Above 40		
	0	1	0.99	0.06
	Less technology proficient	More technology proficient		
	0	1	**2.82**	0.06
SN	Males	Females		
	0	1	0.19	0.04
	40 and below	Above 40		
	0	1	−0.70	0.04
	Less technology proficient	More technology proficient		
	0	1	**2.73**	0.04
PI	Males	Females		
	0	1	−1.26	0.06
	40 and below	Above 40		
	0	1	−1.40	0.06
	Less technology proficient	More technology proficient		
	0	1	0.41	0.06
SI	Males	Females		
	0	1	0.54	0.06
	40 and below	Above 40		
	0	1	−1.03	0.06
	Less technology proficient	More technology proficient		
	0	1	1.15	0.06
PBC	Males	Females		
	0	1	−1.63	0.04
	40 and below	Above 40		
	0	1	**4.40**	0.05
	Less technology proficient	More technology proficient		
	0	1	−1.16	0.06
SE	Males	Females		
	0	1	−0.79	0.04
	40 and below	Above 40		
	0	1	1.52	0.04
	Less technology proficient	More technology proficient		
	0	1	**7.65**	0.04
RFC	Males	Females		
	0	1	−0.32	0.06
	40 and below	Above 40		
	0	1	1.09	0.06
	Less technology proficient	More technology proficient		
	0	1	**5.80**	0.06
TFC	Males	Females		
	0	1	−0.57	0.05
	40 and below	Above 40		
	0	1	–**2.08**	0.05
	Less technology proficient	More technology proficient		
	0	1	**8.83**	0.05
Intentions	Males	Females		
	0	1	−1.85	0.04
	40 and below	Above 40		
	0	1	0.54	0.04
	Less technology proficient	More technology proficient		
	0	1	1.74	0.05

*Critical ratios (CR) >+/– 1.96 are highlighted in bold*.

Latent mean comparisons between males and females revealed no significant differences between genders. However, latent mean comparisons between working adults 40 years and below vs. working adults above 40 years old revealed that the latter had significantly higher PBC ratings than the former (CR = 4.40, *SE* = 0.05, *p* < 0.001). In contrast, working adults above 40 years had significantly lower ratings on TFC than working adults 40 years and below (CR = −2.08, *SE* = 0.05, *p* = 0.037). Lastly, of the 12 factors, only five factor means (i.e., Attitude, PI, SI, PBC, and intentions) were invariant by less technologically proficient individuals and highly technologically proficient individuals. Latent mean comparisons between those who were less proficient in technology vs. those who were highly proficient in technology showed that adults who were highly proficient in technology also rated significantly higher on PU (CR = 2.22, *SE* = 0.06, *p* = 0.027), PEU (CR = 5.70, *SE* = 0.06, *p* < 0.001), Compatibility (CR = 2.82, *SE* = 0.06, *p* = 0.005), SN (CR = 2.73, *SE* = 0.04, *p* = 0.006), SE (CR = 7.65, *SE* = 0.04, *p* < 0.001), RFC (CR = 5.80, *SE* = 0.06, *p* < 0.001), and TFC (CR = 8.83, *SE* = 0.05, *p* < 0.001). These findings are discussed in greater detail in the discussion.

### Structural Invariance

As various tests of measurement invariance have been satisfied, we proceeded to test the structural invariance (i.e., factor variance and covariance invariance) of our model. The entire variance and covariance matrix was constrained to be invariant for each group across gender, age, and technology proficiency; this test of equality was found tenable across gender as overall model fit was still acceptable despite a ΔCFI of 0.011 (Model 5A), but untenable across age (Model 5B; ΔCFI = 0.012, SRMR = 0.09 [ΔSRMR = 0.011]), and technology proficiency (Model 5C; ΔCFI = 0.016, SRMR = 0.09). Our findings imply that the structural relationships among various latent constructs were invariant across gender, but varied depending on one's age and level of technology proficiency. Overall, these findings indicated that while measurement invariance existed in our data, structural invariance did not (except across gender). In other words, subsequent analyses testing the equivalence of each structural parameter in each group across age and technology proficiency would not be contaminated by differences associated with measurement artefacts (Chen et al., 2005).

### Regression Path Differences

Following the rejection of the test of structural invariance across age and technology proficiency level, each structural parameter (or regression path) was tested independently for structural invariance by constraining one path to be invariant across groups while freeing all other paths (see Table 5).

**Table 5:** Regression path differences.

	***df* (Δ*df)***	***χ*^2^(Δ*χ*^2^ from base model)**	***p*** **- value**	**CFI (ΔCFI)**	**SRMR (ΔSRMR)**	**RMSEA(ΔRMSEA)**	**Decision**
***Age group−40 years and below (N** **=** **395) and above 40 years (N** **=** **233)***							
Base model(all paths freely estimated)	1,234	2,808.79	–	0.907(–)	0.085 (–)	0.064(–)	**–**
PU → ATT	1,235 (1)	2,809.73(0.95)	0.331	0.907(0)	0.085(0)	0.064(0)	Accept
PEU → ATT	1,235 (1)	2,808.82(0.03)	0.865	0.907(0)	0.085(0)	0.064(0)	Accept
COMP → ATT	1,235 (1)	2,811.98(3.19)	0.074	0.906(0.001)	0.085(0)	0.064(0)	Accept
PI → SN	1,235 (1)	2,809.47(0.69)	0.408	0.907(0)	0.085(0)	0.064(0)	Accept
SI → SN	1,235 (1)	2,810.55(1.76)	0.184	0.907(0)	0.085(0)	0.064(0)	Accept
SE → PBC	1,235 (1)	2,810.13(1.35)	0.246	0.907(0)	0.085(0)	0.064(0)	Accept
RFC → PBC	1,235 (1)	2,814.79(6.00)	0.014	0.906(0.001)	0.085(0)	0.064(0)	Accept
TFC → PBC	1,235 (1)	2,814.03(5.24)	0.022	0.906(0.001)	0.085(0)	0.064(0)	Accept
ATT → INT	1,235 (1)	2,816.12(7.34)	0.007	0.906(0.001)	0.085(0)	0.064(0)	Accept
SN → INT	1,235 (1)	2,809.50(0.71)	0.399	0.907(0)	0.085(0)	0.064(0)	Accept
PBC → INT	1,235 (1)	2,811.85(3.06)	0.080	0.906(0.001)	0.085(0)	0.064(0)	Accept
***Technology proficiency—Moderately proficient and below (N** **=** **204) and highly proficient and above (N** **=** **424)***							
Base model(all paths freely estimated)	1,234	2,692.40	–	0.910(–)	0.085 (–)	0.061(–)	**–**
PU → ATT	1,235 (1)	2,692.50(0.09)	0.764	0.910(0)	0.085(0)	0.064(0)	Accept
PEU → ATT	1,235 (1)	2,692.51(0.10)	0.748	0.910(0)	0.085 (0)	0.064(0)	Accept
COMP → ATT	1,235 (1)	2,692.45(0.05)	0.830	0.910(0)	0.085(0)	0.064(0)	Accept
PI → SN	1,235 (1)	2,694.27(1.87)	0.172	0.910(0)	0.085(0)	0.064(0)	Accept
SI → SN	1,235 (1)	2,693.82(1.41)	0.235	0.910(0)	0.085(0)	0.064(0)	Accept
SE → PBC	1,235 (1)	2,692.42(0.01)	0.913	0.910(0)	0.085(0)	0.064(0)	Accept
RFC → PBC	1,235 (1)	2,695.83(3.42)	0.064	0.910(0)	0.085(0)	0.064(0)	Accept
TFC → PBC	1,235 (1)	2,695.67(3.27)	0.071	0.910(0)	0.085(0)	0.064(0)	Accept
ATT → INT	1,235 (1)	2,692.98(0.57)	0.500	0.910(0)	0.085(0)	0.064(0)	Accept
SN → INT	1,235 (1)	2,693.14(0.73)	0.392	0.910(0)	0.085(0)	0.064(0)	Accept
PBC → INT	1,235 (1)	2,692.65(0.24)	0.621	0.910(0)	0.085(0)	0.064(0)	Accept

*ATT, Attitudes; PU, Perceived usefulness; PEU, Perceived ease of use; COMP, Compatibility; SN, Subjective norms; PI, Peer influence; SI, Superior influence; PBC, Perceived behavioural control; RFC, Resource facilitating conditions; TFC, Technology facilitating conditions; INT, Intentions*.

Although the test of structural invariance across age and technology proficiency levels was not supported, our findings mainly revealed no significant differences in path coefficients between working adults 40 years and below and those above 40 years, and between those who were less proficient in technology and those who were highly proficient in technology. Assessing the difference between the base model nested within a more constrained model with a χ^2^ test of difference revealed three structural paths (RFC → PBC, TFC → PBC, and ATT → INT) that were significantly different from the base model at an alpha level of 0.05. However, based on a Bonferroni-corrected critical *p* value (0.05/11 regression paths = 0.004) to account for the multiple comparisons we made and possibly inflated type I error rates, and changes in other fit indices such as CFI, SRMR, and RMSEA (i.e., ΔCFI ≤ 0.01, ΔRMSEA ≤ 0.015, ΔSRMR ≤ 0.01), none of these three structural path differences reached statistical significance. Therefore, we conclude that although there were slight differences in path coefficients between those of different age groups, none of these differences were statistically significant; in other words, all paths were invariant by both groups.

## Discussion and Implications

The present study examined the validity of the DTPB model for explaining intentions to participate in microlearning amongst working adults. We were specifically interested in assessing if constructs of the DTPB model were invariant across groups. As working professionals constitute a heterogeneous population, this study examined measurement (configural, metric, scalar, strict) and structural (factor variance and covariance) invariance to assess if latent constructs and relationships in the DTPB model were equivalent across different demographic groups (i.e., gender, age, and technology proficiency level). Establishing measurement invariance across groups would allow for meaningful comparisons to be made about the relationships among constructs, or about the latent constructs themselves, which potentially translates into practical significance for facilitating and accelerating microlearning adoption with the workforce.

Firstly, results of this study revealed that a 12-factor model was valid in explaining microlearning usage intentions of all working adults, regardless of demographic differences. Next, our findings showed that measurement invariance (configural, metric, scalar, and strict) was supported for each group across gender, age, and technology proficiency, and thus allowed for subsequent comparisons of latent mean differences across groups. Following the satisfaction of the various tests of measurement invariance, we attempted to establish structural invariance (factor variance and covariance) for each group across gender, age, and technology proficiency. We found that while measurement invariance existed in our data, structural invariance was only found across gender, not age and technology proficiency; the structural model imposed on various latent constructs was invariant across gender, but varied depending on one's age and level of technology proficiency. However, further analysis revealed that none of the structural path differences reached statistical significance.

### Microlearning Usage Intentions - Gender Differences

In terms of gender, we found no significant differences in the factors explaining microlearning usage intentions amongst males and females. This is perhaps one most surprising finding of our study as it contradicts previous work that has often found both normative beliefs and PBC being more salient for women than men (Morris and Venkatesh, 2000; Venkatesh et al., 2000). Venkatesh et al. (2000) also found that the influence of attitude on intentions to use technology was more salient for men. However, a literature search revealed that several authors have similarly failed to find gender differences in technology adoption contexts. For example, Baker et al. (2007) used the TPB with over a thousand Saudi Arabian workers to investigate moderating effects of gender on adoption of new technology, but failed to find a significant interaction between gender and behaviour intentions. One reason provided by the authors, that may also apply in our research, was that previous research showing moderating effects of gender on intentions to use technology often used a Western sample; as such, their findings may not generalise to a non-Western survey sample. Further research using TPB and DTPB in non-Western cultures are necessary to ascertain the validity of this claim.

The current study provides another plausible reason for null gender differences in behavioural intentions. To date, only a handful of studies investigating an intentions-based model have tested the assumption of measurement invariance prior to making comparisons across different participant types (e.g., Teo, 2015, 2019). Perhaps the failure to control for measurement invariance led to significant gender differences resulting from the combination of both measurement-related differences and between-group differences. In other words, differences found do not necessarily represent true differences in path coefficients attributed to group membership. For this reason, we reiterate the importance of having measurement invariance be a prerequisite to testing structural coefficients across groups.

### Microlearning Usage Intentions - Age Differences

In terms of age, as compared to working adults 40 years and below, we found that those above 40 years of age had significantly higher PBC. In other words, those above 40 years perceive themselves as having more control over decisions of participating in microlearning. Although only a limited number of technology usage studies have tested age as a moderator, our findings are in line with previous research that has found the effect of PBC increasing with age. Morris and Venkatesh (2000) tested technology adoption decisions using the TPB and reported that PBC was more salient for older workers. Hill et al. (2015) reported that older adults above 55 years of age sometimes see mastering new digital technology as a way to empower themselves in this age of digitalisation. Thus, this might be one reason why PBC increases with age. Furthermore, testing for measurement invariance at least helps us reject measurement-related differences as a reason for finding differences between older and younger working adults. However, since other factors (e.g., job seniority) were not controlled for, it is difficult to determine if the reason for this difference is simply a result of *age*.

Despite higher perceptions of control, those above 40 have significantly lower ratings on TFC. This implies that working adults above 40 years also believed that the compatibility of technology and technical assistance would be less than ideal to support their use of microlearning. Similarly, Morris and Venkatesh (2000) found an increased importance being placed on technical assistance to support the use of new technology with increasing age. Therefore, we recommend that when introducing new technology and encouraging the use of technology with working adults, more resources and support should be made available to older working adults.

Although tests of structural invariance were not supported across age groups—while some path coefficients were different between working adults 40 years and below, and those above 40 years, none of these differences reached statistical significance. In other words, the relationships among latent variables were equally applicable for both older and younger individuals.

### Microlearning Usage Intentions - Technology Proficiency Differences

In terms of technology proficiency, only five factor means (i.e., Attitude, PI, SI, PBC, and Intentions) were invariant by less technologically proficient individuals and highly technologically proficient individuals. With all other factors, individuals with lower technology proficiency were significantly lower on the scale. Despite being similar with regards to intentions to participate in microlearning, individuals who rated themselves as being less technologically proficient perceived microlearning as more difficult to use, not as useful, less compatible with their learning style, were less influenced by opinions of others, less confident in participating in microlearning, and believed the resources and technological support available to them were lacking. This highlights an important consideration when introducing microlearning, or any new technology, to working adults; despite its advantages and benefits, a substantial number of individuals will be less technologically proficient than others and require more support.

Knight (2015) recommends customising efforts to integrate and introduce new technology based on workers' level of familiarity with and interest in technology. For example, an online training session may be sufficient for some workers, while others may require more guidance and handholding in the form of a coach or trainer. Understanding the technological proficiency and comfort level of one's employees, then appropriately managing feelings of uncertainty toward new changes, buffers against resistance against new technology and prevents underutilisation.

We may speculate that due to lower perceptions of PU, PEU, compatibility, SN, PBC, RFC, and TFC in those less technologically proficient, some path coefficients may also be different between the two groups. However, none of the differences in the relationships among latent variables between those who were less proficient in technology and those who were highly proficient in technology reached statistical significance. Again, this implies that, the relationships among latent variables were equally applicable for both individuals of lower and higher technological proficiency levels. This may be somewhat surprising, considering some previous findings of moderating effects of technology proficiency on general technology usage behaviour. For instance, Choi et al. (2019) noted that participants' self-reported lack of proficiency in technology was one major contributing factor to their decreased perseverance in using a new application system.

However, it must also be noted that participants in this study were only asked one question regarding their proficiency in technology, making it far from a comprehensive measure of overall technology proficiency. Additionally, with ~95% of the participants identifying themselves as moderately proficient in technology or better, compared to the ~5% who rated themselves slightly proficient or not proficient at all in technology, there is a clear underrepresentation of participants with truly low technology proficiency. To this end, it is still too early to state whether both measurement and structural invariance holds for actual low technologically proficient populations. One possible reason for this is due to COVID-19 restrictions which forbade us from conducting physical data collection; our sample may be skewed toward working adults who are better versed with digital technology (e.g., accessing the survey online through a web link).

### Contributions of Current Study

The recent years have seen microlearning gain increasing promise and popularity with professional learning and development. A review of microlearning trends for professional learning noted that just-in-time instruction or on-demand training, some features of microlearning, are particularly valuable in today's fast-paced and digital world (Leong et al., 2020). In order to increase adoption of microlearning use in working professionals, and ensure that efforts to implement microlearning are fruitful, the current study explored the factors that influence intentions to participate in microlearning of various working adult groups.

The present study substantively extends our understanding of working adults' intentions to engage in microlearning, and further probes how factors differently influence one's behavioural intentions between males and females, between older and younger adults, and between less technologically proficient and more technologically proficient individuals. By using a multigroup invariance analysis, which entailed successive tests of more restrictive models to establish that our measurement instrument being used operates in the same way across different groups (i.e., measurement invariance), we were then able to test for latent mean differences and regression path differences between groups following support for measurement invariance in our data. Few studies, if any, have conducted measurement and structural invariance tests for intentions-based models with the working population.

Considering the heterogeneity of this population in terms of age, gender, and technology proficiency, we demonstrated the importance of understanding how different groups can vary on the same factors of the DTPB. For instance, as compared to older working adults, younger adults perceived themselves as having lesser control over decisions of participating in microlearning (i.e., lower average PBC). They were also no more likely to believe they had control over participating in microlearning even with the required resources needed to participate available to them, while the availability of resources was a significant predictor of older working adults' perceptions of control for engaging in microlearning. Moving forward, it is thus essential to address and account for such differences in any microlearning implementation efforts in the workplace and in adult learning institutions.

### Limitations and Future Research

Although we explored differences in behavioural intentions to use microlearning for different age groups, gender, and technology proficiency levels, several important covariates were not accounted for and thus we are unable to conclude with certainty that differences found in behavioural intentions occurred merely due to an individual's age, gender, or proficiency with technology. For example, the moderating effects of age (i.e., higher PBC in older working adults) may be explained by an individual's level of seniority held in an organisation. Therefore, future research is necessary to narrow down the explanations for why differences in behavioural intentions were found for different age groups.

Likewise, we did not control for similarities in certain clusters of working adults; for instance, working adults nested within the same job industry are inherently similar to a certain extent, and may produce industry-specific differences in terms of age, gender, and technology proficiency. Although our study collected data on participants' job industry, it served a descriptive purpose and thus there were large differences in the number of working adults from different industries. Future studies could consider ensuring an adequate sample size from each major industry to account for multilevel influences in each group across age, gender, and technology proficiency.

Lastly, a crucial limitation of the current study was the absence of a measure of actual microlearning usage. Despite past evidence showing significant intentions-behaviour link, future studies should replicate this study using measures of actual behaviour as the dependent variable to ascertain the strength of the relationship between behavioural intentions and actual microlearning use behaviour amongst working adults.

## Conclusion

As microlearning gains promise and popularity for use in working professionals' continued learning and development, research into understanding the factors that drive intentions to use microlearning and barriers to adoption, is crucial to ensure implementation efforts are not wasted. Noteworthily, by assessing measurement and structural invariance across different groups of working adults, this study improves our understanding of the relative efficacy of various factors (e.g., PBC, PEU, SN) in predicting intentions to use microlearning, and suggests that considerable differences exist among these groups of working adults.

## Data Availability Statement

The datasets presented in this study can be found here: https://doi.org/10.21979/N9/NZ8N7M.

## Ethics Statement

The studies involving human participants were reviewed and approved by the Nanyang Technological University, Singapore and was carried out under ethical guidelines. The patients/participants provided their written informed consent to participate in this study.

## Author Contributions

SP and MB carried out data collection. SP analysed the data and wrote the manuscript with support for the latter from MB, and feedback from CL and EK. CL and EK supervised the project. All authors contributed substantially to the design, conceptualisation of the study, reviewed the paper, and approved the final version of the manuscript.

## Funding

This study was fully funded by the Workforce Development Agency (WDA), SkillsFuture Singapore (SSG), with the project No. WDARF_IAL_GA18-05, through Institute for Adult Learning (IAL) and Centre for Research and Development in Learning (CRADLE), Nanyang Technological University, Singapore.

## Conflict of Interest

The authors declare that the research was conducted in the absence of any commercial or financial relationships that could be construed as a potential conflict of interest.

## Publisher's Note

All claims expressed in this article are solely those of the authors and do not necessarily represent those of their affiliated organizations, or those of the publisher, the editors and the reviewers. Any product that may be evaluated in this article, or claim that may be made by its manufacturer, is not guaranteed or endorsed by the publisher.

## Footnotes

^1^Decisions about how participants from different age group and technology proficiency level are divided and compared were made post data collection. Rationale behind these data-informed splits is explained in more detail later.

^2^Participants were only asked to identify the age group they belonged to. The age groups are as follows: 17–29 years (1), 30–39 years (2), 40–49 years (3), 50–59 years (4), 60–69 years (5), 70 years and above (6).
