# Supplementary material for: Working Adults' Intentions to Participate in Microlearning: Assessing for Measurement Invariance and Structural Invariance
Source: Front Psychol. 2021 Nov 29;12:759181. doi: 10.3389/fpsyg.2021.759181 (PMC8666600; doi:10.3389/fpsyg.2021.759181)
Supplement: Supplementary file 1 [file Table_1.docx]

# Appendix A

Microlearning Survey

| Factor | Item | Statement |
| --- | --- | --- |
| ATT | x6 | To participate in microlearning is a good idea. |
|  | x7 | I look forward to participating in microlearning. |
|  | x8 | Participating in microlearning is appealing. |
| PU | x9 | It is beneficial for me to participate in microlearning. |
|  | x10 | There is value for me to participate in microlearning. |
|  | x11 | Participating in microlearning increases my productivity at work. |
|  | x12 | Participating in microlearning enhances my efficiency at work. |
| PEU | x13 | It is easy for me to participate in microlearning. |
|  | x14 | I participate in microlearning because it is accessible. |
|  | x15 | I think that participating in microlearning is easy. |
|  | x16 | To participate in microlearning is difficult. |
| COMP | x17 | To participate in microlearning fits the way I learn. |
|  | x18 | Participating in microlearning is something I can get used to. |
|  | x19 | I think participating in microlearning fits the way I learn. |
|  | x20 | I would participate in microlearning as it is compatible with the way I learn. |
| SN | x21 | People who are important to me would think that I should participate in microlearning. |
|  | x22 | People who influence my behaviour would think that I should participate in microlearning. |
|  | x23 | People whose opinions I value would think that I should participate in microlearning. |
|  | x24 | I am expected to participate in microlearning. |
| PI | x25 | Peers who are important to me expect me to participate in microlearning. |
|  | x26 | Peers who influence my behaviour would think that I should participate in microlearning. |
|  | x27 | ~~I will participate in microlearning if my peers have done/are doing so too.~~  The opinions of my peers, for whether I participate in microlearning, is important to me. |
|  | x28 | I feel pressured by my peers to participate in microlearning. |
| SI | x29 | The people in higher management, that influence my behaviour, would think that I should participate in microlearning. |
|  | x30 | My superior, whom I report to, would expect me to participate in microlearning. |
|  | x31 | The people in higher management expect that I participate in microlearning. |
|  | x32 | There is an expectation from my superior, whom I report to, for me to participate in microlearning. |
| PBC | x33 | I am able to choose to participate in microlearning or not. |
|  | x34 | I am confident that if I wanted to, I could participate in microlearning. |
|  | x35 | I am in control of whether or not I participate in microlearning. |
|  | x36 | Whether or not I participate in microlearning is completely up to me. |
| SE | x37 | I could easily participate in microlearning on my own. |
|  | x38 | I know enough about how to participate in microlearning. |
|  | x39 | I am confident of participating in microlearning even if I have never used it before. |
|  | x40 | I am confident of participating in microlearning even if there is nobody around to show me how. |
| RFC | x41 | I can participate in microlearning anywhere that is connected to the internet. |
|  | x42 | The resources (e.g. money, time, internet connection) needed to participate in microlearning are available to me. |
|  | x43 | I could easily get access to the resources (e.g. money, time, internet connection) that are needed to participate in microlearning. |
|  | x44 | I have the equipment (e.g. computer, laptop, smartphone, tablet etc.) to participate in microlearning. |
| TFC | x45 | The technology (e.g. web apps, mobile apps) to participate in microlearning is something I am familiar with. |
|  | x46 | I think there is enough technical support for me to participate in microlearning. |
|  | x47 | I know there will be technical assistance if I encounter technical difficulties while participating in microlearning. |
|  | x48 | I do not shy away from participating in microlearning due to possible technical difficulties. |
| INT | x49 | I intend to spend time participating in microlearning. |
|  | x50 | I intend to make an effort to participate in microlearning. |
|  | x51 | I will participate in microlearning in the future. |

*Note.* ATT = Attitudes, PU = Perceived usefulness, PEU = Perceived ease of use, COMP = Compatibility, SN = Subjective norms, PI = Peer influence, SI = Superior influence, PBC = Perceived behavioural control, SE = Self-efficacy, RFC = Resource facilitating conditions, TFC = Technology facilitating conditions, INT = Intentions.
